# Distinct small-fiber dysfunction profiles in CMT1A and RFC1 disease: a multimodal study

**DOI:** 10.1007/s00415-026-14145-w

**Published:** 2026-09-16

**Authors:** Sara Massucco, Massimo Leandri, Viola Bruzzone, Lucilla Nobbio, Mehrnaz Hamedani, Silvia Stara, Emilia Bellone, Alessandro Geroldi, Consuelo Venturi, Chiara Gemelli, Angelo Schenone, Lucio Marinelli, Marina Grandis

**Affiliations:** 1 Department of Neuroscience, Rehabilitation, Ophthalmology, Genetics, Maternal and Child Health (DINOGMI), University of Genoa, Genoa, Italy; 2 AOM - IRCCS Ospedale Policlinico San Martino, Clinica Neurologica, Genoa, Italy; 3 AOM - IRCCS Ospedale Policlinico San Martino, Division of Clinical Neurophysiology and Epilepsy Center, Genoa, Italy; 4 AOM - IRCCS Ospedale Policlinico San Martino, Pathology Unit, Genoa, Italy

**Keywords:** CMT1A, CANVAS, RFC1, Small fiber neuropathy, Pain, Skin biopsy

## Abstract

**Background:**

Charcot-Marie-Tooth disease 1A (CMT1A) and Cerebellar Ataxia, Neuropathy, Vestibular Areflexia Syndrome (CANVAS)/Replication Factor Complex subunit 1 (RFC1)-related disease affect large nerve fibers, but small fiber dysfunction may contribute to pain and dysautonomia. We applied a multimodal protocol, including potentials elicited by a micropatterned electrode targeting intraepidermal nerve endings, to characterize small fiber involvement.

**Methods:**

In this cross-sectional study, healthy controls (HC) and patients with CMT1A or RFC1 disease underwent neurologic examination, autonomic assessment with the composite autonomic symptom score−31 (COMPASS-31) and compound autonomic dysfunction test (CADT), electrochemical skin conductance (ESC), nociceptive evoked potentials (NEPs), pain-related evoked potentials (PREPs), somatosensory evoked potentials (SEPs), and skin biopsy.

**Results:**

Twenty-one patients with CMT1A, 16 with RFC1 disease, and 21 HC were included. Neuropathic pain occurred in 33% of CMT1A and 81% of RFC1 patients. Dysautonomia was prominent in RFC1 disease, with abnormal CADT scores in 94%, COMPASS-31 scores of 7–46, and abnormal ESC in 63%, compared with 24% in CMT1A. N40 NEP latencies were prolonged in both patient groups versus HC (p<0.001), with absent responses in 33% and 44%, respectively. PREPs showed prolonged N2 latencies in CMT1A (p=0.003), with absent N2 responses in 57%; in RFC1 disease, N2 responses were absent in 50%. Skin biopsy showed length-dependent intraepidermal nerve fiber density loss in CMT1A and severe non-length-dependent epidermal denervation in RFC1 disease.

**Discussion:**

Multimodal assessment integrating ESC, NEPs, PREPs, and skin biopsy identifies distinct Aδ- and C-fiber dysfunction patterns in CMT1A and RFC1 disease and may support phenotyping and small-fiber biomarker development in peripheral neuropathies.

**Supplementary Information:**

The online version contains supplementary material available at https://doi.org/10.1007/s00415-026-14145-w.

## Introduction

Small fiber dysfunction is increasingly recognized as a clinically relevant yet undercharacterized component of several hereditary neuropathies. Beyond large fiber impairment, involvement of small myelinated Aδ fibers and unmyelinated C fibers may contribute to neuropathic pain, thermal sensory deficits, and autonomic symptoms, but remains difficult to capture with a single diagnostic tool.

Charcot-Marie-Tooth disease type 1 A (CMT1A) is the most common hereditary sensorimotor neuropathy and results from a 1.5-Mb duplication on chromosome 17p encompassing the *peripheral myelin protein 22* (*PMP22*) gene [1–3]. While traditionally classified as a demyelinating neuropathy, CMT1A is better conceptualized as a dysmyelinating disorder caused by abnormal myelin development, characterized by uniform slowing of nerve conduction across peripheral nerves [4, 5]. Large myelinated fibers are predominantly affected; however, growing evidence indicates involvement of Aδ and C fibers [6–9].

Biallelic intronic AAGGG pentanucleotide repeat expansions in the *replication factor C subunit 1* (*RFC1*) gene are a frequent cause of cerebellar ataxia, neuropathy, and vestibular areflexia syndrome (CANVAS) and related late-onset ataxias [10]. The prevalence of RFC1 disease is likely underestimated, given the relatively high carrier frequency of pathogenic repeat expansions and the identification of additional pathogenic motifs [11, 12]. Most affected individuals develop late-onset sensory neuronopathy with variable vestibular and cerebellar involvement, and repeat expansion size may influence disease onset and progression [12–14]. Even if ataxia is the predominant clinical feature of RFC1 disease, autonomic symptoms, neuropathic pain, and thermal sensory deficits are frequently reported [14–17]. Sural nerve morphometry demonstrates marked loss of large myelinated fibers, accompanied by reduction of small myelinated and unmyelinated fibers, with minimal or no evidence of regeneration [18, 19]. Skin biopsy studies have consistently shown severe intraepidermal denervation [15, 17, 18, 20], and some reports have also documented autonomic involvement and sudomotor dysfunction [15–17, 21, 22].

Skin biopsy is an established structural marker of small fiber loss, enabling quantification of intraepidermal nerve fiber density (IENFD). However, assessment of small fiber function remains more challenging, as reliable neurophysiological tools are limited. Sudoscan provides a rapid, noninvasive measure of sudomotor function through electrochemical skin conductance (ESC), reflecting chloride ion flux in response to a low-voltage direct current applied to the skin [23]. ESC abnormalities have been described in several hereditary neuropathies, particularly hereditary transthyretin amyloidosis [24–28], and have also been explored in RFC1 disease [15, 17, 22].

More recently, a surface electrode with a micropatterned interdigitated design and inter-rail gap of 150 μm (150 IDE) has been developed for selective stimulation of intraepidermal nerve endings [29]. Random interval stimulation of the dorsum of the hand using the 150 IDE allows recording of pain-related evoked potentials (PREPs), with latencies similar to those obtained with laser stimulation [29]. Sustained rhythmic stimulation with the 150 IDE also elicits early lateralized cortical responses, with the first negative component (N40) thought to reflect activation of primary somatosensory cortex via fast-conducting Aδ fibers [28, 30, 31]. Because of their purely nociceptive nature, devoid of endogenous late components, they are referred to as nociceptive evoked potentials (NEPs) [28, 30].

Given the limitations of any single technique, integrating structural, neurophysiological, and autonomic measures may better capture the full spectrum of small fiber dysfunction. This study aimed to apply such a multimodal approach to characterize small fiber involvement in CMT1A and RFC1 disease.

## Methods

### Study population and patient selection

Participants included individuals with genetically confirmed CMT1A or RFC1 disease, as well as healthy controls. Patients were identified among all individuals attending our referral center during the study period. This included both patients with a previously established diagnosis who were evaluated during routine follow-up visits and patients who received a new diagnosis following assessment at our center. All consecutive patients who met the predefined inclusion and exclusion criteria were enrolled.

For inclusion in the CMT1A group, patients were required to have a genetically confirmed heterozygous duplication of the 17p11.2–p12 region encompassing the *PMP22* gene. For inclusion in the RFC1 disease group, patients were required to have a compatible clinical phenotype and genetically confirmed biallelic pathogenic pentanucleotide repeat expansions in intron 2 of the *RFC1* gene. Healthy controls were recruited among unaffected family members of patients and volunteers from the hospital staff.

Individuals with other potential causes of or risk factors for peripheral neuropathy, including diabetes mellitus, paraproteinemia, hypothyroidism, vitamin deficiencies, or alcohol consumption exceeding two drinks per day, were excluded.

### Study design

This was a single-center, cross-sectional study. Demographic and clinical variables collected included age, sex, age at symptom onset, disease duration, neurological manifestations, and ongoing treatment for neuropathic pain. Patients underwent a multimodal evaluation including: (1) neurological examination with clinical scales, including the Charcot-Marie-Tooth Examination Score (CMTES), the Scale for the Assessment and Rating of Ataxia (SARA), and the Douleur Neuropathique 4 (DN4) questionnaire; (2) assessment of orthostatic hypotension, with brachial blood pressure measured at baseline and at 60 and 180 s after standing, along with evaluation of autonomic symptoms using the Compound Autonomic Dysfunction Test (CADT) and the Composite Autonomic Symptom Score−31 (COMPASS-31); (3) electrochemical skin conductance (ESC) measurement; (4) recording of NEPs and PREPs; (5) sensory nerve conduction studies and somatosensory evoked potentials (SEPs); and (6) skin biopsy for IENFD quantification. Healthy participants underwent NEP, PREP, and SEP recordings and ESC measurement for comparison.

NEPs and PREPs were elicited using the same peripheral nociceptive stimulus but different stimulation paradigms to assess complementary stages of nociceptive processing. NEPs focused on the early contralateral N40 response, reflecting the early cortical arrival of predominantly Aδ-mediated nociceptive input, whereas PREPs assessed later N1 and N2-P2 event-related cortical responses, which reflect subsequent endogenous cortical processing and are more susceptible to modulation by attention, stimulus salience, and habituation [28, 30]. Thus, both techniques were included to characterize early and later cortical processing of the same nociceptive afferent input.

To ensure consistency across assessments, all ESC measurements were performed by a single trained operator (S.M.), whereas neurophysiological recordings were acquired by three trained operators (L.M., M.L., and S.M.), all of whom followed the same standardized acquisition protocol. The physiological domains and principal nerve fiber populations evaluated by each assessment method are summarized in Table 1.

**Table 1:** Summary of autonomic, neurophysiological, and morphological assessments used in the study

Assessment method	Fiber type and/or function evaluated	Main outcome reported
CADT	Autonomic symptom burden across orthostatic, gastrointestinal, and genitourinary domains (patient-reported)	Total score (% of maximum score)
COMPASS-31	Multidomain autonomic symptom burden (patient-reported)	Total weighted score
ESC (Sudoscan)	Sudomotor function, providing an indirect index of postganglionic sympathetic cholinergic function	Hand and foot ESC values (μS)
NEPs	Early cortical response to predominantly Aδ-mediated nociceptive afferent input	N40 latency, amplitude, and response recordability
PREPs	Later event-related cortical responses reflecting processing of predominantly Aδ-mediated nociceptive input	N1 and N2 latencies, amplitudes, and response recordability
Sensory NCS	Peripheral conduction along large myelinated sensory fibers, predominantly Aβ fibers	SNAP amplitude and sensory conduction velocity
SEPs	Conduction along large myelinated somatosensory afferents, predominantly Aβ fibers, and central dorsal column–medial lemniscal pathways	N20 latency and response recordability
Skin biopsy	Structural integrity of intraepidermal small somatic sensory fibers, including Aδ and C fibers	IENFD (fibers/mm)

*CADT* compound autonomic dysfunction test, *COMPASS-31* composite autonomic symptom score−31, *ESC* electrochemical skin conductance, *IENFD* intraepidermal nerve fiber density, *NCS* nerve conduction studies, *NEPs* nociceptive evoked potentials, *PREPs* pain-related evoked potentials, *SEPs* somatosensory evoked potentials, *SNAP* sensory nerve action potential

### Clinical scales and questionnaires

Neuropathy severity was assessed using the CMTES (range 0–28, higher scores indicating greater severity) [32]. In RFC1 disease, ataxia severity was measured with the SARA scale (range 0–40, higher scores indicating more severe ataxia) [33]. Autonomic symptoms were evaluated using the COMPASS-31 (range 0–100, higher scores indicating greater symptom burden) and the CADT (maximum score 20 in males and 16 in females, lower scores indicating more severe autonomic dysfunction) [34, 35]. To account for sex-related differences in CADT scoring, results were expressed as percentages [28]. Neuropathic pain was defined as a DN4 score of 4 or higher [36].

### NEPs

Previously published stimulation and recording procedures were followed [28]. The 150 IDE (15 × 15 mm; Bionen S.A.S., Florence, Italy) was applied to the hairy skin of the dorsum of the dominant hand, between the first and second metacarpal bones. Stimulation consisted of bursts of 10 electrical pulses, each lasting 0.2 ms, delivered at 1-ms intervals, with intensity set at 1.5 times the perception threshold. Stimuli were delivered at 0.83 Hz for 500–1000 repetitions. Impedance was maintained between 16 and 30 kΩ in a temperature-controlled room.

Neural responses were recorded using subdermal electrodes placed at C3’/C4’-Fz. Signals were amplified ×100,000, band-pass filtered (0.1–2000 Hz), and acquired over an 800-ms time window (300 ms prestimulus, 500 ms poststimulus) at a sampling rate of 25,000 samples/s.

### PREPs

The stimulation site and electrode configuration were identical to those used for NEPs and previously described [28]. However, the temporal stimulation paradigm was modified to elicit later pain-related cortical responses while minimizing habituation. Only 30 stimuli were delivered at randomized intervals of 5–14 s, at an intensity of 1.5 times the perception threshold [28]. After each stimulus, participants rated perceived intensity on a numerical scale from 0 (no sensation) to 10 (maximum sensation), targeting a rating of 2–3.

Lateralized N1 responses were recorded at C3'/C4'-Fz, whereas the vertex N2-P2 complex was recorded at Cz-Au1/2.

### SEPs

Radial SEPs were recorded maintaining the same stimulation site, intensity, and rate described for NEPs, but using self-adhesive Ag/AgCl electrodes, as previously described [28]. Median nerve SEPs were obtained by stimulating the median nerve at the wrist using single 0.2-ms pulses at motor threshold intensity.

Cortical responses were recorded using subdermal electrodes at C3'/C4'-Fz.

### Conventional nerve conduction studies

Sensory electroneurography of the radial nerve was performed using the standard antidromic technique. Recordings were obtained using surface electrodes placed on the hairy skin at the base of the thumb, with the active electrode positioned proximally and the reference electrode distally. Electrical stimulation was delivered proximally at the dorsolateral aspect of the wrist using a bipolar stimulator. Supramaximal rectangular pulses with a duration of 0.2 ms were applied to ensure consistent activation of sensory fibers. The inter-electrode distance was carefully measured to accurately calculate the conduction velocity. Skin temperature was maintained above 32 °C to avoid variability in latency and amplitude. The signals were amplified, filtered (band-pass: 20 Hz to 2 kHz), and averaged over multiple trials to improve the signal-to-noise ratio. Sensory nerve action potentials (SNAPs) were analyzed in terms of onset latency, onset-to-peak amplitude, and conduction velocity. Throughout the procedure, care was taken to minimize artefacts and ensure proper electrode contact.

Additional motor and sensory nerve conduction data were obtained from routine clinical assessments, when available.

### Quantification of ESC changes

Sudomotor function was assessed using a SUDOSCAN 2 device (Impeto Medical, Paris, France; software version 3.2.3601). Examinations were performed in a temperature-controlled room maintained at 22–24 °C. Participants stood barefoot with the soles of both feet positioned centrally on the foot electrodes and the palms of both hands placed flat on the hand electrodes. They were instructed to remain still and maintain complete contact with the electrodes throughout the approximately 3-min examination. After automatic calibration, the device applied a low-voltage direct current and automatically measured ESC for each hand and foot, expressed in microSiemens (μS). ESC values ≥60 μS for the hands and ≥70 μS for the feet were considered normal [37].

### Skin biopsy

Skin samples were collected using a 3-mm disposable punch under sterile conditions following topical anesthesia with lidocaine. Samples were collected from the distal lateral leg (approximately 10 cm proximal to the lateral malleolus) and the distal lateral thigh (approximately 15 cm above the patella). Tissue processing followed internationally standardized protocols [38] and previously described methods [28, 39]. Specimens were fixed overnight at 4 °C in 2% paraformaldehyde-lysine periodate solution, cryoprotected, and sectioned with a freezing microtome. Free-floating 50-µm sections were incubated with a primary rabbit antibody targeting the pan-neuronal marker protein gene product 9.5, followed by a biotinylated anti-rabbit IgG secondary antibody. Detection was performed using the avidin–biotin-peroxidase complex with a chromogenic substrate. IENFD was quantified at 40× magnification using light microscopy. At least three 50-µm sections per biopsy were analyzed. IENFD values were expressed as fibers per millimeter and classified as abnormal when below the age- and sex-adjusted 5th percentile [40]. The pathologist (A.S.) was blinded to clinical diagnosis.

### Data analysis

Continuous variables are reported as medians with ranges or interquartile ranges (IQR) or as means with standard deviations (SDs). Categorical variables are presented as absolute counts and percentages. Normality was assessed using the Shapiro–Wilk test, and homogeneity of variance using Levene’s test. General linear models were used to assess the effects of diagnosis (CMT1A, RFC1 disease, or controls) and age on neurophysiological measures, with diagnosis entered as a fixed factor and age as a covariate. Heteroscedasticity-consistent standard errors (HC3) were applied to account for violations of model assumptions. Bonferroni-adjusted pairwise comparisons were derived from the same age-adjusted models.

Associations between clinical scales, autonomic questionnaires, neurophysiological measures, and IENFD were examined using Pearson or Spearman correlation coefficients as appropriate.

Statistical significance was defined as a two-sided *p*-value <0.05. Because normative values for NEPs and PREPs obtained with the 150 IDE are not available, patient latencies were compared with the mean latency of healthy controls. Latencies exceeding the control mean by more than 2 SDs were classified as prolonged. Amplitudes were considered abnormal only when responses were absent. Analyses were performed using jamovi (version 2.4.28) and R (version 4.5.0) within RStudio.

## Results

### Demographic and clinical characteristics of participants

A total of 21 healthy controls (8 males, 13 females; median age, 51 years [IQR, 42–65; range, 30–82]), 21 patients with CMT1A (8 males, 13 females; median age, 46 years [IQR, 31–58; range, 22–66]), and 16 patients with RFC1 disease (12 males, 4 females; median age, 68.5 years [IQR, 62–73.25; range, 57–85]), were included. All patients with RFC1 disease harbored biallelic expansions of the AAGGG pentanucleotide repeat motif in intron 2 of the *RFC1* gene.

Patients presented with varying degrees of neuropathy severity, with CMTES ranging from 1 to 21 (median, 7) in CMT1A and from 3 to 17 (median, 10.5) in RFC1 disease. SARA scores ranged from 1 to 25 (median, 9.5) in patients with RFC1 disease.

**Fig. 1:**
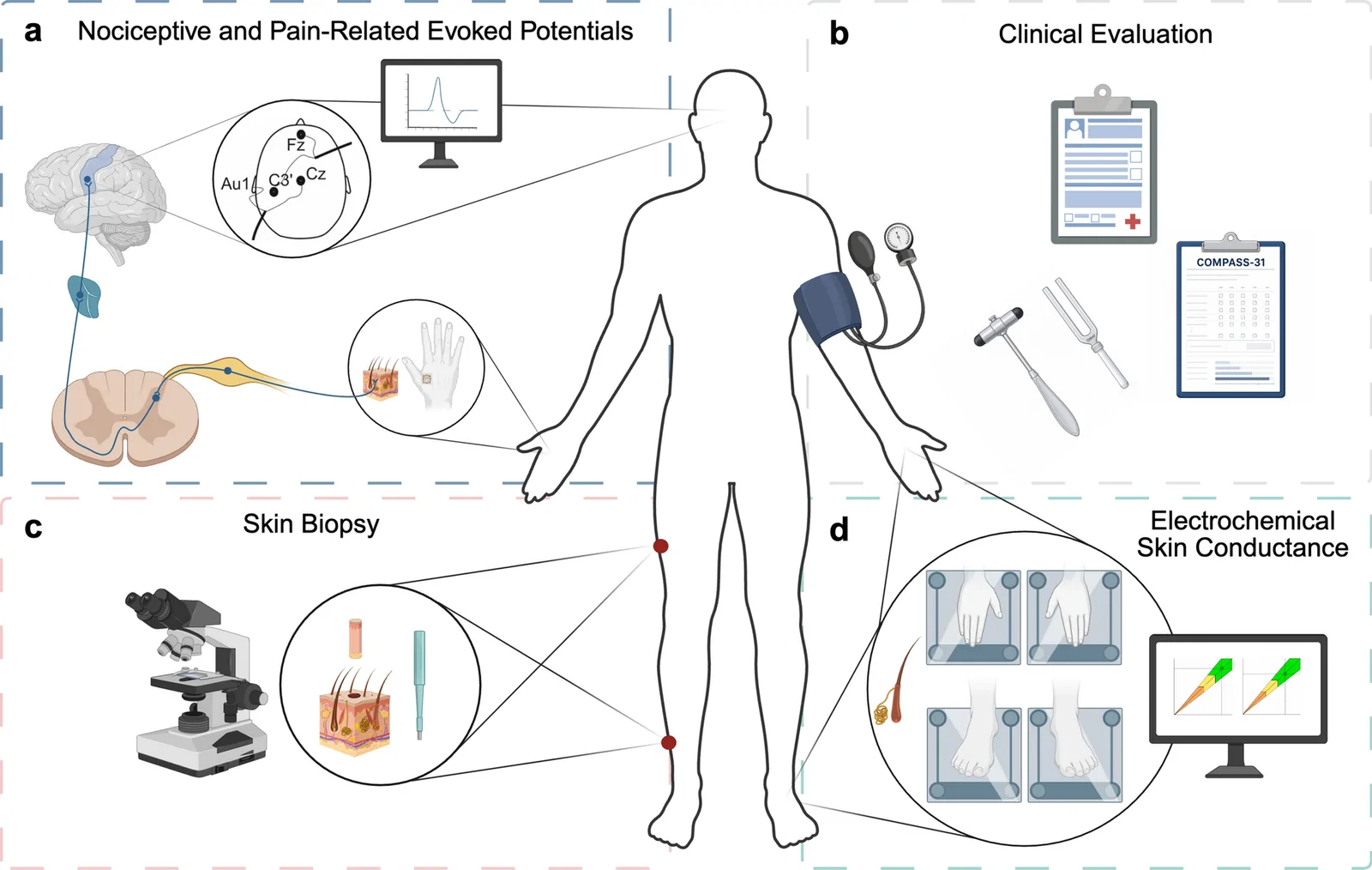
Multimodal protocol for the assessment of small-fiber involvement. **a** Nociceptive and pain-related evoked potentials are obtained after stimulation with the micropatterned interdigitated electrode (150 IDE) applied to the dorsum of the hand, with cortical responses [N40 (C3′–Fz), N1 (C3′–Fz), and N2 (Cz–A1/A2) components] recorded from scalp electrodes. **b** Clinical assessment includes neurological examination and clinical rating scales, including the Composite Autonomic Symptom Score−31 (COMPASS-31). **c** Skin punch biopsy is performed at the distal leg and distal thigh for the quantification of intraepidermal nerve fiber density. **d** Electrochemical skin conductance is measured at the palms and soles using Sudoscan as an index of sudomotor function. Created in BioRender. Geroldi, A. (2026) https://BioRender.com/kcl2ts9

Demographic and clinical characteristics are summarized in Table 2, whereas Fig. 1 illustrates the multimodal evaluation protocol.

**Table 2:** Demographic, clinical characteristics, and intraepidermal nerve fiber density of patients with CMT1A and RFC1 disease

	CMT1A (*n* = 21)	RFC1 Disease (*n* =16)	Healthy Controls (*n* = 21)
Age, median (range) (years)	46 (22–66)	68.5 (57–85)	51 (30–82)
Males (n, %)	8, 38.1%	12, 75.0%	8, 38.1%
Disease duration, median (IQR) (years)	25 (12–31)	10.50 (5.75–18.25)	-
Chronic cough			-
n, %	-	16, 100%	
Age of onset, median (IQR) (years)		39.31 ± 11.18	
Neuropathic pain (n, %)	7, 33.33%	13, 81.3%	-
Pain features (n, %)			-
Burning	4/7, 57.1%	7/13, 53.9%	
Electric shock-like	1/7, 14.3%	2/13, 15.4%	
Pinprick	2/7, 28.6%	4/13, 30.8%	
Constrictive	0, 0%	2/13, 15.4%	
Stabbing	0, 0%	1/13, 7.7%	
Allodynia	0, 0%	3/13, 23.1%	
Itching (n, %)	0, 0%	1, 6.25%	-
Pain distribution (n, %)			-
Distal limbs, symmetric	7/7, 100%	10/13, 76.9%	
Diffuse	0, 0%	3/13, 23.1%	
Neuropathic pain therapy (n, %)			-
Pregabalin	3/7, 42.9%	6/13, 46.2%	
Gabapentin	0, 0%	2/13, 15.4%	
Duloxetine	0, 0%	6/13, 46.2%	
Combination (pregabalin or gabapentin plus duloxetine)	0, 0%	4/13, 30.8%	
CMTES (0–28), mean ± SD	8.19 ± 5.02	11.5 ± 4.24	-
SARA (0–40), mean ± SD	-	10.31 ± 7.24	-
CADT (% of total), median (IQR)	100 (85–100)	69.38 (54.69–76.25)	-
COMPASS-31 (0–100), mean ± SD	8.62 ± 7.11	21.25 ± 11.70	-
IENFD, median (IQR) (fibers/mm)			
Leg IENFD	2.60 (1.73–3.20)	1.00 (0.40–1.15)	-
Thigh IENFD	5.17 (3.81–6.73)	0.40 (0.30–2.21)	

*CADT* compound autonomic dysfunction test, *CMT1A* Charcot-Marie-Tooth disease type 1 A, *CMTES* Charcot-Marie-Tooth examination score, *COMPASS-31* composite autonomic symptom score−31, *IENFD* intraepidermal nerve fiber density, *IQR* interquartile range, *RFC1* Replication Factor C subunit 1, *SARA* scale for the assessment and rating of ataxia, *SD* standard deviation

Neuropathic pain (DN4 score ≥4) was present in 7 patients with CMT1A (33.3%) and 13 with RFC1 disease (81.3%). In CMT1A, pain typically followed a length-dependent pattern. In RFC1 disease, pain was also generally symmetrical and length-dependent; however, 23.1% of patients reported diffuse allodynia involving the head and trunk as well (Table 2). Two patients with RFC1 disease had experienced burns due to impaired heat perception.

Most CMT1A patients reported only mild autonomic symptoms, mainly xerophthalmia, xerostomia, and skin color changes. Twelve (57.1%) had a CADT score of 100%, and the mean COMPASS-31 score was 8.62±7.11. In contrast, 15 of 16 patients with RFC1 disease (93.8%) exhibited reduced CADT scores, and COMPASS-31 scores ranged from 7 to 46 (median, 20). The most commonly reported symptoms were orthostatic intolerance (68.8%), diarrhea or constipation (68.8%), and sphincter dysfunction (62.5%), notably urinary incontinence or retention; one patient required intermittent catheterization. Some patients with RFC1 disease reported near-syncope on standing, and one experienced an episode of postprandial syncope. Three patients reported delayed gastric emptying with frequent episodes of emesis. Three others had severe constipation: 2 underwent surgery for intestinal volvulus at ages 63 and 67, whereas the third had abnormal rectal manometry findings and required pyridostigmine, laxatives, and enemas. COMPASS-31 scores correlated with disease severity as measured by the SARA score (r = 0.511; 95% CI, 0.021–0.803; *p* = 0.043).

Conventional nerve conduction findings are summarized in Online Resource 1. Studies showed a demyelinating pattern in CMT1A, with marked slowing of motor and sensory conduction velocities, together with reduced or absent sensory responses. In contrast, RFC1 disease showed a predominantly sensory axonal pattern, with reduced or absent sensory responses and largely preserved motor conduction velocities.

### ESC measurement

Five CMT1A patients (23.8%) and 10 RFC1 patients (62.5%) had reduced ESC values (Online Resource 2).

In general linear models, diagnosis significantly influenced ESC at all sites (right hand: *p* < 0.001, η^2^p = 0.292; left hand: *p*<0.001, η^2^p = 0.300; right foot: *p* = 0.003, η^2^p = 0.187; left foot: *p* = 0.004, η^2^p = 0.206), whereas age had only minor effects. Post-hoc analyses showed higher ESC values in controls compared with both CMT1A and RFC1 disease at the hands (all *p* ≤ 0.001), with no difference between CMT1A and RFC1 disease. At the feet, ESC values were lower in RFC1 patients than in controls bilaterally (all *p* ≤ 0.021), whereas CMT1A patients differed from controls only at the right foot (*p* = 0.049); no differences were observed between the two patient groups (Table 3).

**Table 3:** Neurophysiological and sudomotor measures in patients with RFC1 disease, patients with CMT1A, and healthy controls

Measure			CMT1A (*n* = 21)	RFC1 (*n* = 16)	Controls (*n* = 21)	*p*-value (age-adjusted)	Post-hoc (Bonferroni, age-adjusted)
NEPs							
N40		Latency (ms)	59.91 ± 8.94	46.18 ± 2.75	40.87 ± 1.14	<0.001^***^	CMT1A – RFC1 (*p* < 0.001^***^) CMT1A – Controls (*p* < 0.001^***^) RFC1 – Controls (*p* < 0.001^***^)
		Amplitude (µV)	0.28 ± 0.28	0.43 ± 0.49	0.93 ± 0.63	<0.001^***^	CMT1A – RFC1 (*p* = 0.736) CMT1A – Controls (*p* < 0.001^***^) RFC1 – Controls (*p* = 0.068)
PREPs							
N1		Latency (ms)	148.17 ± 16.08	136.35 ± 6.02	137.93 ± 19.52	0.410	CMT1A – RFC1 (*p* = 0.604) CMT1A – Controls (*p* = 0.680) RFC1 – Controls (*p* = 1.000)
		Amplitude (µV)	4.57 ± 5.14	3.72 ± 5.66	9.26 ± 6.53	0.012^*^	CMT1A – RFC1 (*p* = 0.565) CMT1A – Controls (*p* = 0.009^**^) RFC1 – Controls (*p* = 0.287)
N2		Latency (ms)	252.97 ± 38.70	205.25 ± 21.21	202.43 ± 44.97	<0.001^***^	CMT1A – RFC1 (*p* < 0.001^***^) CMT1A – Controls (*p* = 0.003^**^) RFC1 – Controls (*p* = 1.000)
		Amplitude (µV)	1.46 ± 2.09	3.80 ± 6.44	6.92 ± 7.08	0.009^**^	CMT1A – RFC1 (*p* = 0.124) CMT1A – Controls (*p* = 0.007^**^) RFC1 – Controls (*p* = 1.000)
Radial SEPs							
N20		Latency (ms)	36.27 ± 9.64	26.90 ± 5.20	20.91 ± 0.93	<0.001^***^	CMT1A – RFC1 (*p* = 0.030^*^) CMT1A – Controls (*p* < 0.001^***^) RFC1 – Controls (*p* = 0.004^**^)
		Amplitude (µV)	0.75 ± 0.53	0.80 ± 0.43	1.44 ± 2.09	0.341	CMT1A – RFC1 (*p* = 1.000) CMT1A – Controls (*p* = 0.590) RFC1 – Controls (*p* = 0.432)
Perception threshold for the 150 IDE							
Threshold (mA)			1.55 ± 0.54	1.90 ± 0.51	1.29 ± 0.52	0.017^*^	CMT1A – RFC1 (*p* = 0.824) CMT1A – Controls (*p* = 0.396) RFC1 – Controls (*p* = 0.020^*^)
ESC values							
Right hand (µS)			70.10 ± 10.75	53.31 ± 22.10	79.05 ± 4.87	<0.001^***^	CMT1A – RFC1 (*p* = 0.233) CMT1A – Controls (*p* = 0.001^**^) RFC1 – Controls (*p* < 0.001^***^)
Left hand (µS)			70.05 ± 10.07	55.50 ± 20.78	79.76 ± 4.88	<0.001^***^	CMT1A – RFC1 (*p* = 0.394) CMT1A – Controls (*p* < 0.001^***^) RFC1 – Controls (*p* < 0.001^***^)
Right foot (µS)			77.52 ± 7.99	59.56 ± 25.22	81.47 ± 4.56	0.003^**^	CMT1A – RFC1 (*p* = 0.255) CMT1A – Controls (*p* = 0.049^*^) RFC1 – Controls (*p* = 0.021^*^)
Left foot (µS)			78.19 ± 7.76	59.38 ± 23.56	81.19 ± 5.18	0.004^**^	CMT1A – RFC1 (*p* = 0.109) CMT1A – Controls (*p* = 0.126) RFC1 – Controls (*p* = 0.010^*^)

*150 IDE* interdigitated electrode with rail gaps of 150 µm, *CMT1A* Charcot-Marie-Tooth disease type 1 A, *ESC* electrochemical skin conductance, *NEPs* nociceptive evoked potentials, *PREPs* pain-related evoked potentials, *RFC1* Replication Factor C subunit 1-related disease, *SEPs* somatosensory evoked potentials

Values are expressed as mean ± standard deviation. Overall* p* values and Bonferroni-adjusted pairwise comparisons were obtained from age-adjusted general linear models

^*^p < 0.05

^**^p < 0.01

^***^p < 0.001

ESC values correlated with autonomic measures. Lower CADT scores, indicating greater autonomic dysfunction, were associated with lower foot ESC (right foot: ρ = 0.489, *p* = 0.002; left foot: ρ = 0.519, *p* = 0.001). Higher COMPASS-31 scores, reflecting greater autonomic symptom burden, were associated with lower foot ESC values (right foot: ρ = − 0.375, *p* = 0.022; left foot: ρ = − 0.373, *p* = 0.023).

In RFC1 disease, lower ESC values were associated with greater ataxia severity, reflected by higher SARA scores (hands: ρ = − 0.597, *p* = 0.015; feet: ρ = − 0.501, *p* = 0.048).

### NEPs

Stimulation intensity was maintained below 5.00 mA in all participants, consistently eliciting a localized pinprick sensation. Higher perception thresholds with the 150 IDE were associated with greater neuropathy severity, as reflected by higher CMTES scores (ρ = 0.455, *p* = 0.005). Perception thresholds are detailed in Table 3.

In the CMT1A group, N40 responses were absent in 7 patients (33.3%), while the remaining 14 (66.7%) showed prolonged N40 latencies exceeding 3 SDs above the mean of healthy controls.

Among patients with RFC1 disease, N40 responses were non-recordable in 7 (43.8%), and 8 (50%) exhibited prolonged N40 latencies exceeding 2 SDs above the control mean (Online Resource 2).

N40 latencies showed relatively low variability, particularly in controls (SD, 1.14 ms; Table 3), indicating stable early nociceptive responses across participants.

**Fig. 2:**
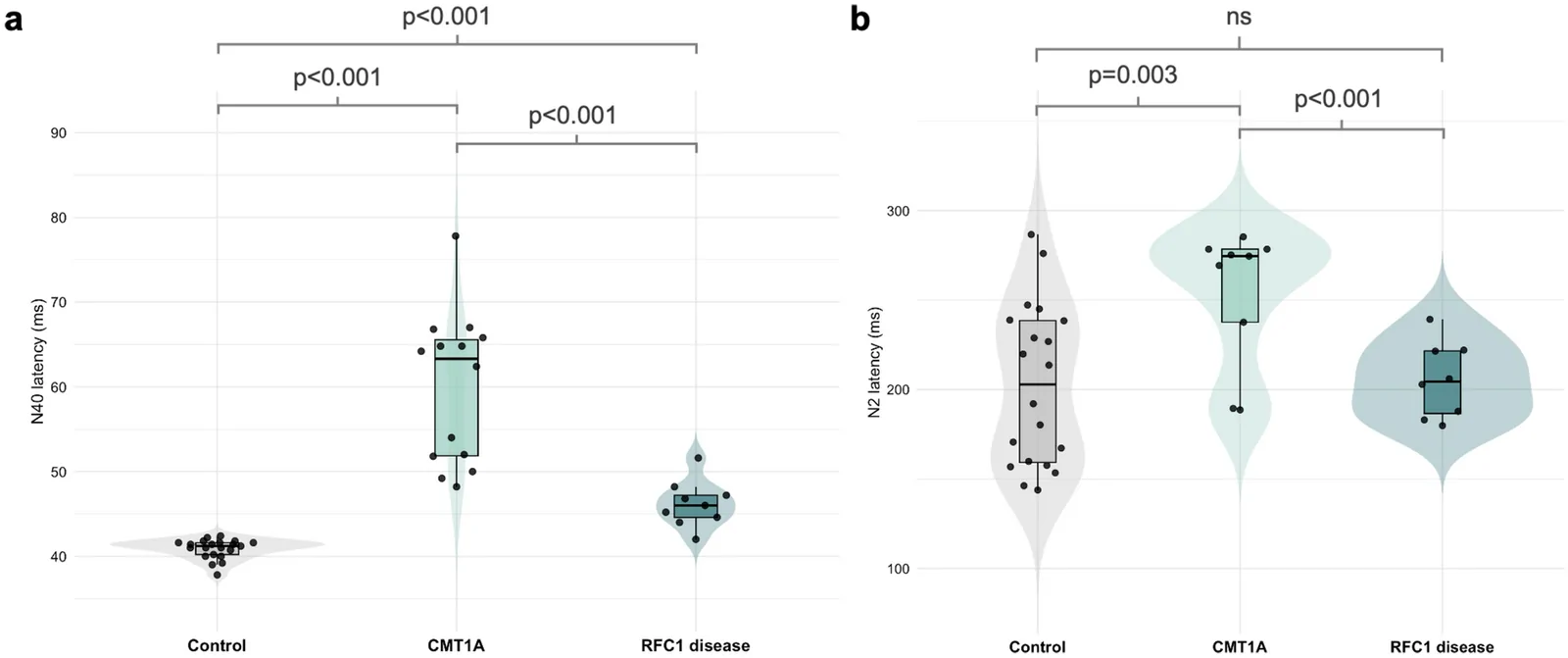
Latencies of nociceptive and pain-related evoked potentials. **a** Comparison of N40 latencies from nociceptive evoked potentials between controls, patients with CMT1A, and patients with RFC1 disease. **b** Comparison of N2 latencies from pain-related evoked potentials between controls, patients with CMT1A, and patients with RFC1 disease. Each point represents one participant. Only participants with recordable nociceptive or pain-related evoked potentials were included in these comparisons; differences between the number of plotted points and the total sample size reflect unrecordable responses. Reported p-values are adjusted for age and obtained from generalized linear models; ns = not significant

A general linear model adjusted for age demonstrated a strong effect of diagnosis on N40 latency (*p *< 0.001, η^2^p = 0.720), whereas age was not a significant contributor (*p* = 0.720). Post-hoc comparisons showed longer N40 latencies in CMT1A compared with both RFC1 disease (*p* < 0.001) and controls (*p* < 0.001); RFC1 patients also differed from controls (*p* = 0.001; Table 3, Fig. 2).

N40 amplitudes differed across groups (*p* < 0.001, η^2^p = 0.275) but were not influenced by age (*p* = 0.491). Age-adjusted comparisons showed reduced N40 amplitudes in CMT1A compared with controls (*p* <0.001), whereas RFC1 patients showed intermediate values, not significantly different from either controls (*p* = 0.068) or CMT1A (*p* = 0.736; Table 3).

### PREPs

Following random-interval stimulation, 12 patients with CMT1A (57.1%) exhibited absent vertex N2 responses; of these, 9 also lacked lateralized N1 responses (Online Resource 2). Among patients with RFC1 disease, N1 responses were non-recordable in 8 (50%), and N2 responses were absent in 8 (50%; Online Resource 2). Compared with NEPs, PREP measures showed greater variability across all groups, including healthy controls. In particular, N1 and N2 latencies displayed wider dispersion (controls: N1 SD, 19.52 ms; N2 SD, 44.97 ms).

N1 latency did not differ significantly across diagnostic groups after adjustment for age (*p* = 0.410) (Table 3). N1 amplitudes were influenced by both diagnosis (*p* = 0.012, η^2^p = 0.179) and age (*p* = 0.016, η^2^p = 0.118). Age-adjusted analyses showed reduced N1 amplitudes in CMT1A compared to controls (*p* = 0.009), whereas RFC1 patients showed intermediate values not significantly different from either group (Table 3).

For N2 latencies, both diagnosis (*p* < 0.001, η^2^p = 0.379) and age (*p* = 0.004, η^2^p = 0.218) were significant contributors. Post-hoc comparisons revealed prolonged N2 latencies in CMT1A compared with both RFC1 disease (*p* < 0.001) and controls (*p* = 0.003), whereas RFC1 patients did not differ from controls (Table 3). N2 amplitudes were also influenced by diagnosis (*p* = 0.009, η^2^p = 0.211) but not by age (*p* = 0.051). Post-hoc analyses demonstrated reduced N2 amplitudes in CMT1A compared with controls (*p* = 0.007), with no significant differences between RFC1 disease and the other groups (Table 3).

### Skin biopsy results

Skin biopsies were obtained from 13 patients with CMT1A at the distal leg and, in a subset of 6, also at the distal thigh. Twelve of 13 patients (92.3%) had leg IENFD values below the 5th percentile for age and sex.

**Fig. 3:**
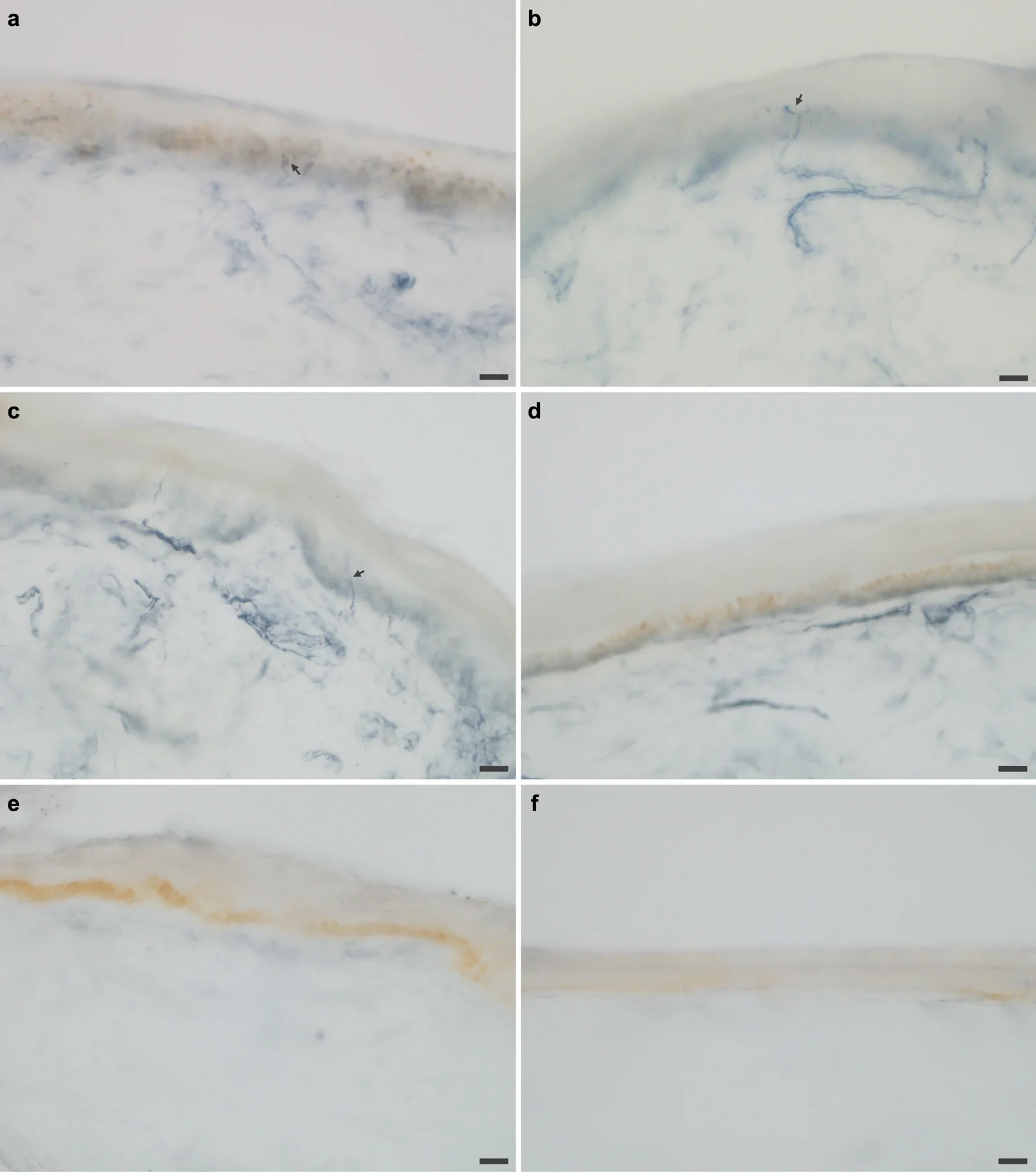
Representative skin biopsy images from a healthy control and patients with CMT1A and RFC1 disease **a** Healthy control, distal thigh. **b** Healthy control, distal leg. **c** CMT1A, distal thigh. **d** CMT1A, distal leg. **e** RFC1 disease, distal thigh. **f** RFC1 disease, distal leg. Arrows indicate examples of intraepidermal nerve fibers crossing the dermal–epidermal junction. In CMT1A, intraepidermal denervation is more pronounced at the distal leg than at the thigh, consistent with a predominantly length-dependent pattern. In RFC1 disease, complete intraepidermal denervation is evident at both the distal thigh and distal leg, consistent with a severe non-length-dependent pattern. Skin biopsies were evaluated by bright-field immunohistochemistry using an antibody against the pan-neuronal marker protein gene product 9.5 (PGP9.5). All images were acquired using a 40× objective. Scale bars, 20 μm

Thirteen patients with RFC1 disease underwent biopsy at both the distal leg and the distal thigh. All but one showed severe intraepidermal denervation, with a median IENFD of 1.0 fibers/mm at the distal leg and 0.4 fibers/mm at the distal thigh. Representative skin biopsy images from the distal thigh and distal leg of a healthy individual, a patient with CMT1A, and a patient with RFC1 disease are shown in Fig. 3.

One patient developed burning dysesthesia in the distal limbs and diffuse allodynia at age 57. Conventional nerve conduction studies were normal, but skin biopsy performed at age 60 revealed reduced IENFD. Given unremarkable laboratory findings, the patient was initially diagnosed with idiopathic small fiber neuropathy. At age 62, sural sensory nerve action potential amplitudes became mildly reduced, and subsequent genetic testing identified biallelic AAGGG intronic expansions in intron 2 of *RFC1*, confirming the diagnosis of RFC1 disease.

## Discussion

The present study provides converging clinical, neurophysiological, and morphological evidence that small nerve fibers are involved in both CMT1A and RFC1 disease, but with substantially different patterns. In CMT1A, small fiber abnormalities were predominantly distal and consistent with the length-dependent distribution of peripheral nerve involvement characteristic of the disease. By contrast, RFC1 disease was associated with severe small fiber involvement extending to proximal sites and prominent painful and autonomic manifestations, supporting a non-length-dependent process consistent with the underlying sensory neuronopathy. Rather than simply demonstrating small fiber dysfunction in these disorders, our findings suggest that the anatomical distribution and clinical expression of small fiber involvement differ between CMT1A and RFC1 disease, reflecting distinct underlying pathophysiological mechanisms.

This contrast was also evident in the pattern of neuropathic pain. In CMT1A, neuropathic pain was distal and symmetric, paralleling the length-dependent pattern of intraepidermal denervation. Pain is common in CMT1A but may be of different types: previous studies have reported overall pain in up to 88% of patients, whereas neuropathic pain accounted for a substantially smaller proportion, approximately 18% in one well-characterized cohort [6, 41–45]. Thus, beyond confirming the high overall prevalence of pain in CMT1A, our findings support a clinically relevant neuropathic component in a subset of patients (one-third in our cohort), whose distal symmetric distribution parallels the length-dependent pattern of small fiber pathology. In RFC1 disease, neuropathic pain was considerably more prominent and occasionally extended beyond a length-dependent distribution, with diffuse allodynia involving the trunk, head, and limbs. The prevalence observed in our cohort was higher than that reported in a recent systematic review, which estimated a pooled prevalence of neuropathic pain of 43.2%, although with substantial inter-study variability [46]. More importantly, the non-length-dependent distribution of pain in some patients paralleled the widespread intraepidermal denervation observed histologically. This clinical–morphological concordance is consistent with a sensory neuronopathy, rather than a conventional distal axonopathy, characterized by involvement of different populations of primary sensory neurons and neuronal loss predominantly in the dorsal root and cranial sensory ganglia [47]. Diffuse allodynia might additionally reflect altered processing of nociceptive input following sensory deafferentation, although this remains speculative and the mechanisms generating neuropathic pain in RFC1 disease require further investigation. The occurrence of clinically relevant thermal sensory loss, including accidental burns in two patients, further emphasizes that small fiber dysfunction in RFC1 disease may have consequences extending beyond positive sensory symptoms.

Chronic cough may represent another manifestation of widespread sensory involvement in RFC1 disease and can precede the onset of ataxia by several years or even decades [13]. Although its pathophysiology remains incompletely understood, accumulating evidence suggests that cough may reflect early dysfunction of sensory afferent pathways rather than merely accompany the neurological phenotype. Patients with biallelic pathogenic *RFC1* expansions and apparently isolated refractory chronic cough have already been shown to exhibit widespread sensory neuron involvement, suggesting that peripheral sensory dysfunction may precede the development of the full neurological phenotype [48]. This interpretation is also consistent with the severe somatic small fiber denervation reported in RFC1 disease, including in patients with relatively short disease duration [17]. The sensory afferent limb of the cough reflex is mediated by vagal Aδ and C fibers projecting to brainstem nuclei, and dysfunction of these pathways has been proposed as a mechanism underlying chronic cough in RFC1 disease [49]. More recently, pathological examination of patients with CANVAS demonstrated marked depletion of bronchial and cutaneous sensory nerve fibers despite heightened cough reflex sensitivity, raising the possibility that denervation-related hypersensitivity contributes to chronic cough [50]. In this context, the long interval between cough onset and other neurological manifestations in our cohort, together with the patient who developed small fiber neuropathy before large-fiber sensory involvement, is compatible with asynchronous vulnerability of different sensory neuronal populations. Chronic cough may therefore represent an early marker of peripheral sensory involvement within the RFC1 disease spectrum. However, our study did not directly assess vagal afferents, and a causal relationship between chronic cough and selective involvement of vagal small fibers cannot be established from these data.

The contrast between CMT1A and RFC1 disease was also evident in autonomic impairment. Autonomic manifestations were generally mild in CMT1A, whereas RFC1 disease was associated with clinically relevant dysfunction across several autonomic domains. Orthostatic hypotension was observed in over two-thirds of patients with RFC1 disease and was often asymptomatic, a finding of clinical relevance in individuals with gait instability and an already increased risk of falls. Gastrointestinal disturbances and sphincter dysfunction were also common, sometimes with severe manifestations, including intestinal volvulus and urinary dysfunction requiring catheterization. These findings are consistent with previous studies demonstrating cardiovascular autonomic dysfunction, gastrointestinal and genitourinary involvement, and sudomotor abnormalities in RFC1 disease [15, 17, 21]. Taken together, they suggest that autonomic dysfunction may represent a clinically relevant, although variably expressed, component of the RFC1 phenotype. Pathological studies have nevertheless demonstrated particularly severe and widespread somatic small fiber denervation in RFC1 disease [17], suggesting a possible dissociation in the severity of somatic and autonomic small fiber involvement. Reported rates of sudomotor abnormalities also vary across studies and testing modalities, ranging from ~20% to 42% with Sudoscan [15, 17, 22] to 56% with quantitative sudomotor axon reflex test (QSART) [21]. In our cohort, reduced ESC values were more frequent in RFC1 disease than in CMT1A and were associated with greater ataxia severity; however, given the small sample size and the indirect nature of ESC as a measure of sudomotor function, this association should be considered exploratory. The lower frequency of ESC abnormalities in CMT1A is consistent with previous evidence that autonomic small fibers may also be affected in this predominantly large-fiber demyelinating neuropathy [7, 9, 51]. Across groups, ESC values were generally higher at the feet than at the hands, consistent with site-specific reference distributions and thresholds.

Neurophysiological findings provided complementary evidence of small fiber involvement in both CMT1A and RFC1 disease. NEPs elicited by selective intraepidermal stimulation primarily assess the early cortical response to predominantly Aδ-mediated nociceptive input, with the N40 considered to reflect an early stage of cortical processing following arrival of the afferent volley. In contrast, PREPs are later event-related cortical responses elicited by the same peripheral nociceptive input and reflect subsequent cortical processing, which is more susceptible to modulation by attention, stimulus salience, and habituation [28, 30]. The greater interindividual variability observed for PREP N1 and N2 latencies compared with the relatively consistent N40 latency, including among healthy participants, may reflect the greater influence of higher-order cortical processing on these later responses. Thus, NEPs and PREPs may be regarded as complementary measures of different stages of nociceptive processing. Importantly, the neurophysiological pattern differed between the two disorders. In CMT1A, N40 latency was substantially prolonged compared with RFC1 disease and healthy controls, suggesting prominent slowing along surviving Aδ-mediated nociceptive pathways. Prolonged N2 PREP latencies provided additional evidence of altered later pain processing in CMT1A. This pattern is consistent with previous laser-evoked potential (LEP) studies demonstrating abnormalities of nociceptive responses in CMT1A, including reduced N2-P2 amplitudes [8] and prolonged N2 latencies following foot stimulation [52]. These findings may reflect involvement of thinly myelinated afferents within the broader dysmyelinating neuropathic process. In RFC1 disease, by contrast, NEPs and PREPs were frequently non-recordable, while latency prolongation was less pronounced among the responses that remained recordable. In the context of the severe and widespread somatic small fiber loss characteristic of RFC1 disease [17], this pattern may reflect loss of functioning nociceptive afferents rather than predominant slowing of surviving pathways. However, this interpretation remains inferential, as cortical evoked potentials cannot localize dysfunction exclusively to the peripheral nerve. Moreover, because latency comparisons necessarily include only recordable responses, the relatively preserved latencies observed in RFC1 disease should not be interpreted as evidence of milder nociceptive pathway involvement. Rather, the neurophysiological findings complement the severe structural small fiber loss observed on skin biopsy and previous reports of frequently abnormal LEPs in RFC1 disease [17].

Skin biopsy provided the clearest anatomical distinction between the two disorders. In CMT1A, intraepidermal denervation predominantly affected the distal leg, in agreement with previous reports [9, 53]. The absence of a significant relationship between IENFD and CMTES in our cohort does not necessarily indicate that small fiber loss is unrelated to disease burden, because CMTES predominantly captures manifestations of large-fiber sensorimotor neuropathy and may therefore be relatively insensitive to variation in small fiber pathology. The limited sample size may also have reduced the ability to detect such an association. Conversely, the severe reduction of IENFD at both distal and proximal sites in RFC1 disease closely mirrors previous reports of extreme, non-length-dependent somatic denervation and reinforces the concept that small fiber pathology forms part of the sensory neuronopathy rather than representing a secondary distal neuropathy [15, 17, 18, 20]. The observation that small fiber abnormalities preceded conventional nerve conduction abnormalities in one patient is particularly intriguing in light of a previous report of a similar case with initial isolated small fiber neuropathy [17], possibly suggesting that somatic skin denervation may represent an early marker of RFC1 disease [17]. However, this individual observation cannot establish the temporal sequence of small- and large-fiber involvement and should instead be considered hypothesis-generating for future longitudinal studies.

Taken together, the complementary findings across clinical assessment, skin biopsy, sudomotor testing, and nociceptive evoked potentials support the value of a multimodal approach to small fiber assessment. These techniques provide different and only partially overlapping information, and their combination may therefore be more informative than any single measure in disorders in which small fiber involvement is heterogeneous or coexists with severe large-fiber neuropathy. From a practical perspective, NEPs obtained with the 150-IDE may offer a useful functional assessment using conventional electrophysiological recording equipment. Nevertheless, the absence of established normative reference values currently limits their use as stand-alone diagnostic or monitoring biomarkers, and validation in larger healthy and disease cohorts is needed.

Several limitations should be considered when interpreting these findings. First, the relatively small sample size limits the precision of prevalence estimates and between-group comparisons, reduces the statistical power to detect modest associations, and may affect the generalizability of the observed correlations. The cross-sectional design precludes assessment of the temporal evolution of small fiber abnormalities and their potential relationship with disease progression. Quantitative sensory testing was not performed, precluding direct quantification of thermal detection and pain thresholds. In addition, established normative values for NEP and PREP latencies are currently lacking, and larger studies in healthy individuals across a broader age range are needed. Although between-group comparisons of neurophysiological and ESC measures were adjusted for age, differences in age distribution and sex composition may still have influenced the results, and residual confounding cannot be excluded. Sudoscan also provides an indirect surrogate of sudomotor function rather than a direct measure of postganglionic autonomic C-fiber integrity; therefore, ESC abnormalities should be interpreted in conjunction with clinical, neurophysiological, and morphological findings. Finally, although all patients with RFC1 disease harbored biallelic expansions of the pathogenic AAGGG repeat motif, individual expansion size data were unavailable, preventing assessment of potential genotype–phenotype relationships.

In conclusion, our findings indicate that small fiber involvement is a meaningful component of both CMT1A and RFC1 disease but differs substantially in its distribution and clinical expression. CMT1A was characterized by a predominantly length-dependent pattern of small fiber dysfunction superimposed on the well-established large-fiber dysmyelinating neuropathy, whereas RFC1 disease showed severe non-length-dependent somatic denervation consistent with sensory neuronopathy, together with prominent painful and autonomic manifestations. The complementary structural and functional abnormalities observed support the combined characterization of small fiber involvement using skin biopsy and targeted neurophysiological measures such as NEPs. Larger longitudinal studies are needed to determine whether these measures can identify early disease manifestations or serve as biomarkers of progression in hereditary neuropathies.

## Supplementary Information

Below is the link to the electronic supplementary material.

Supplementary file1 (PDF 96 KB)

Supplementary file2 (PDF 114 KB)

## Data Availability

The data that support the findings of this study are available from the corresponding author upon reasonable request.
