# Supplementary material for: Distinct small-fiber dysfunction profiles in CMT1A and RFC1 disease: a multimodal study
Source: J Neurol. 2026 Sep 16;273(10):602. doi: 10.1007/s00415-026-14145-w (PMC13582087; doi:10.1007/s00415-026-14145-w)
Supplement: Supplementary file 1 [file 415_2026_14145_MOESM1_ESM.pdf]

Online Resource 1: Motor and Sensory Nerve Conduction Studies (*Journal of Neurology*)

Distinct small-fiber dysfunction profiles in CMT1A and RFC1 disease: a multimodal study

Authors

Sara Massucco [1], Massimo Leandri [1], Viola Bruzzone [1], Lucilla Nobbio [2], Mehrnaz Hamedani [1], Silvia Stara [1,3], Emilia Bellone [1], Alessandro Geroldi [1], Consuelo Venturi [4], Chiara Gemelli [2], Angelo Schenone [1,2], Lucio Marinelli [1,3], Marina Grandis [1,2]

Affiliations

[1] Department of Neuroscience, Rehabilitation, Ophthalmology, Genetics, Maternal and Child Health (DINOGMI), University of Genoa, Genova, Italy

[2] AOM - IRCCS Ospedale Policlinico San Martino, Clinica Neurologica, Genova, Italy

[3] AOM - IRCCS Ospedale Policlinico San Martino, Clinical Neurophysiology, Genova, Italy

[4] AOM - IRCCS Ospedale Policlinico San Martino, Pathology Unit, Genova, Italy

Corresponding author: Lucio Marinelli; Department of Neuroscience, Rehabilitation, Ophthalmology, Genetics, Maternal and Child Health (DINOGMI), University of Genoa, and Clinical Neurophysiology, IRCCS Azienda Ospedaliera Metropolitana, Genoa, Italy; e-mail address: lucio.marinelli@unige.it

Table S1. **Motor and sensory nerve conduction study findings in patients with CMT1A and RFC1 disease.**

Variable	Mean ± SD (range)	
	CMT1A (n = 21)	RFC1 disease (n = 16)
dCMAP, recordable, n/N (%)		
Ulnar	21/21 (100%)	12/12 (100%)
Median	1/1 (100%)	8/8 (100%)
Peroneal	2/3 (67%)	16/16 (100%)
Tibial	1/3 (33%)	5/5 (100%)
DML (ms)		
Ulnar	9.83 ± 3.36 (5.20-19.20)	3.18 ± 0.85 (2.00-5.40)
Median	10.30	3.79 ± 0.69 (2.60-4.60)
Peroneal	14.50 ± 2.12 (13.00-16.00)	4.11 ± 0.75 (2.90-5.20)
Tibial	11.60	4.56 ± 1.48 (3.00-7.00)
dCMAP (mV)		
Ulnar	3.36 ± 1.47 (0.20-5.40)	7.39 ± 2.70 (0.70-11.10)
Median	3.70	8.41 ± 3.54 (3.00-12.60)
Peroneal	0.73 ± 0.95 (0.00-1.80)	4.66 ± 1.07 (2.40-7.30)
Tibial	0.30 ± 0.52 (0.00-0.90)	6.58 ± 3.88 (2.00-12.20)
MNCV (m/s)		
Ulnar	21.76 ± 5.23 (13.00-30.00)	55.53 ± 13.57 (24.10-83.00)
Median	23.90	52.43 ± 4.10 (46.00-58.00)
Peroneal	20.50 ± 2.12 (19.00-22.00)	42.74 ± 3.83 (35.00-48.00)
Tibial	16.00	41.64 ± 4.02 (36.00-47.00)
SNAP, recordable, n/N (%)		
Sural	0/3 (0%)	6/16 (38%)
Ulnar	0/3 (0%)	4/11 (36%)
Median	0/1 (0%)	3/10 (30%)
Radial	10/21 (48%)	3/12 (25%)
SNAP (µV)		
Sural	-	1.11 ± 2.34 (0.00-8.60)
Ulnar	-	1.41 ± 2.44 (0.00-6.90)
Median	-	3.00 ± 5.84 (0.00-15.60)
Radial	1.28 ± 1.67 (0.00-4.50)	0.96 ± 1.79 (0.00-4.60)
SNCV (m/s)		
Sural	-	40.83 ± 5.19 (34.00-47.00)
Ulnar	-	55.18 ± 9.46 (42.70-63.00)
Median	-	48.87 ± 3.78 (45.60-53.00)
Radial	21.86 ± 3.53 (18.00-28.00)	49.67 ± 24.25 (23.30-71.00)

Values are presented as mean ± SD (range), unless otherwise specified. Values refer to the non-dominant side. Amplitudes were calculated including absent responses as 0. Distal latencies and conduction velocities were calculated only for recordable responses. For variables with only one available recordable response, the individual value is reported. For motor studies, conduction velocity refers to the principal distal limb segment.
CMT1A = Charcot–Marie–Tooth disease type 1A; dCMAP = distal compound muscle action potential; DML = distal motor latency; MNCV = motor nerve conduction velocity; RFC1 = replication factor C subunit 1; SNAP = sensory nerve action potential; SNCV = sensory nerve conduction velocity.
