# Supplementary material for: Distinct small-fiber dysfunction profiles in CMT1A and RFC1 disease: a multimodal study
Source: J Neurol. 2026 Sep 16;273(10):602. doi: 10.1007/s00415-026-14145-w (PMC13582087; doi:10.1007/s00415-026-14145-w)
Supplement: Supplementary file 2 [file 415_2026_14145_MOESM2_ESM.pdf]

Online Resource 2: Individual Patient Data (*Journal of Neurology*)

Distinct small-fiber dysfunction profiles in CMT1A and RFC1 disease: a multimodal study

Authors

Sara Massucco [1], Massimo Leandri [1], Viola Bruzzone [1], Lucilla Nobbio [2], Mehrnaz Hamedani [1], Silvia Stara [1,3], Emilia Bellone [1], Alessandro Geroldi [1], Consuelo Venturi [4], Chiara Gemelli [2], Angelo Schenone [1,2], Lucio Marinelli [1,3], Marina Grandis [1,2]

Affiliations

[1] Department of Neuroscience, Rehabilitation, Ophthalmology, Genetics, Maternal and Child Health (DINOGMI), University of Genoa, Genova, Italy
[2] AOM - IRCCS Ospedale Policlinico San Martino, Clinica Neurologica, Genova, Italy
[3] AOM - IRCCS Ospedale Policlinico San Martino, Clinical Neurophysiology, Genova, Italy
[4] AOM - IRCCS Ospedale Policlinico San Martino, Pathology Unit, Genova, Italy

Corresponding author: Lucio Marinelli; Department of Neuroscience, Rehabilitation, Ophthalmology, Genetics, Maternal and Child Health (DINOGMI), University of Genoa, and Clinical Neurophysiology, IRCCS Azienda Ospedaliera Metropolitana, Genoa, Italy; e-mail address: lucio.marinelli@unige.it

Table S2. **Skin biopsy and neurophysiological measures in patients with CMT1A and RFC1 disease.**

Pt No.	Thigh IENFD (fibers/mm)	Leg IENFD (fibers/mm)	NEP Latencies (ms)	PREP Latencies (ms)		SEP Latencies (ms)	ESC
			N40	N1	N2	N20	
**CMT1A**							
1	5.9	2.7	**nr**	**nr**	**nr**	**42.7****	n
2	-	-	**nr**	**nr**	**nr**	**nr**	n
3	-	-	**77.8****	**nr**	**nr**	**42.5****	n
4	-	-	**51.8****	162.3	274.5	**24.1****	**red.**
5	-	-	**65.8****	162.1	285.3	**nr**	**red.**
6	-	-	**nr**	**nr**	**nr**	**nr**	n
7	-	-	**nr**	**nr**	**nr**	**nr**	n
8	7	3.2	**50.0****	163.2	269.3	**42.5****	n
9	-	-	**67.0****	**nr**	**nr**	**24.1****	**red.**
10	-	1.9	**nr**	154.4	237.6	**45.7****	**red.**
11	3.6	4.5	**54.0****	**nr**	**nr**	20.1	n
12	2.4	2.6	**49.2****	**nr**	**nr**	**36.5****	n
13	-	-	**52.0****	127.4	188.6	**29.1****	n
14	-	2.5	**64.2****	123.2	**nr**	**35.5****	n
15	-	1.2	**62.4****	148.8	**nr**	**31.3****	**red.**
16	7.8	4.9	**nr**	161.6	278.4	**34.3****	n
17	-	1.3	**64.8****	160.0	189.4	**29.1****	n
18	4.4	1.7	**66.8****	**nr**	**nr**	**37.9****	n
19	-	2.7	**48.2****	156.0	278.4	**34.7****	n
20	-	4.3	**nr**	134.6	**nr**	**51.10****	n
21	-	0.5	**64.8****	124.4	275.2	**55.4****	n
**RFC1 Disease**							
1	3.8	1.3	**nr**	**nr**	183.0	**25.9****	**red.**
2	3.7	4.1	**nr**	**nr**	**nr**	**24.7****	**red.**
3	0.3	0.5	**nr**	**nr**	**nr**	**24.1****	**red.**
4	0.5	0.4	**nr**	**nr**	**nr**	**29.5****	**red.**
5	0.4	0.0	**51.6****	136.8	221.4	**32.5****	n
6	-	-	**45.2****	142.0	206.0	**27.9****	n
7	-	-	**46.8****	**nr**	**nr**	20.5	**red.**
8	0.2	1.0	**nr**	136.6	239.2	**nr**	**red.**
9	2.2	1.2	**nr**	**nr**	**nr**	**41.1****	**red.**
10	-	-	**46.0****	131.4	202.8	**26.9****	n
11	0.3	0.5	**44.6****	138.8	179.8	20.7	n
12	0.4	0.4	**48.2****	142.6	222.0	22.5	n
13	0.4	0.3	**44.0***	124.2	187.8	**24.3****	**red.**
14	4.7	4.0	42.0	138.4	**nr**	**24.5****	n
15	0.0	1.1	**nr**	**nr**	**nr**	**30.1****	**red.**
16	0.3	1.1	**47.2****	**nr**	**nr**	**28.3****	**red.**

CMT1A = Charcot-Marie-Tooth disease type 1A; ESC = electrochemical skin conductance; IENFD = intraepidermal nerve fiber density; NEP = nociceptive evoked potential; PREP = pain-related evoked potential; Pt = patient; RFC1 = replication factor C subunit 1 gene; SEP = somatosensory evoked potentials.

Notes: n = normal; nr = not recordable; * = more than two standard deviations above the mean of healthy controls; ** = more than three standard deviations above the mean of healthy controls; red. = reduced.

SEPs were recorded after stimulation of the dorsum of the dominant hand (radial nerve).
